# Rocksalt-Zincblende–Wurtzite Mixed-Phase ZnO Crystals With High Activity as Photocatalysts for Visible-Light-Driven Water Splitting

**DOI:** 10.3389/fchem.2020.00351

**Published:** 2020-04-29

**Authors:** Titao Li, Haihuai Cai, Caifu Li, Xiaolong Liu, Feng Huang

**Affiliations:** School of Materials, Sun Yat-sen University, Guangzhou, China

**Keywords:** mixed-phase, ZnO photocatalysts, visible light absorption, impurity levels, overall water splitting

## Abstract

Finding out the factors that dominate photocatalytic activity is always an essential topic toward the development of highly active photocatalysts. The increased photoactivity of ZnO:Ga (L) may be attributed to the existence of homojunctions and resultant oxygen vacancies in triphasic ZnO:Ga (L), which can reduce the recombination of photogenerated carriers and provide them higher doping efficiency and higher optical gain. Then, the photocatalytic behaviors of as-prepared N doped crystals have been studied and rationalized to understand the role of each of species played in light absorption and photo activation. The N-doped ZnO:Ga (L) sample which showed higher activity than N-doped ZnO:Ga (B) and ZnO:Ga (L), the high activity could be explained by increase of visible light absorption and presence of empty impurity levels introduced by N doping.

## Introduction

Over the past three decades, photodecomposition of water to hydrogen has been widely studied as a potential application in solar energy conversion to produce hydrogen (Fujishima and Honda, 1972; Zou et al., 2001; Chen et al., 2010; Maeda, 2011). The importance of semiconductors to be used for photocatalysts has spurred a large research effort to understand the fundamentals of photochemical processes and to design new photocatalysts. However, significant challenges remain in developing semiconductor to meet the urgent requirements for photocatalysis so far. The semiconductors with narrow bandgap can absorb sunlight in a wide spectrum but suffer from photo-corrosion during photocatalysis, while the wide bandgap semiconductors have limited light-absorption spectral range although they are optically stable. In order to extend photo responsive range, intense efforts are being focused on doping ions into the wide bandgap semiconductors. Metal oxides with d^0^ orbital such as TiO_2_ and other metal oxides with d^10^ orbitals like ZnO and Ga_2_O_3_ have been investigated extensively, but they have wide band gaps that only allows absorbing ultraviolet (UV) light of <5% of solar radiation (Tafen et al., 2009). Visible light response has been stimulated by nitrogen doping and the presence of N in the structure clearly decreases the band gap. A solid solution of gallium and zinc nitrogen oxide (Ga_1−x_Zn_x_)(N_1−x_O_x_), which is a yellow powder possessing an absorption edge near 510 nm, has received much attention for its highest activity for overall water splitting (Maeda et al., 2005a). These solid solutions were prepared by nitridating mixed powders of Ga_2_O_3_/ZnO in a flowing NH_3_ atmosphere. They have the ZnO crystal structure, with Zn and O isomorphously substituted by Ga and N, respectively. The as-prepared solid solution has negligible photocatalytic activity for overall water splitting. However, the modified solid solution with nanoparticles of a mixed oxide of rhodium and chromium exhibits high activity for overall water splitting to produce H_2_ and O_2_, and this mixture become a promising and efficient photocatalyst. Transition-metal oxide played the role of co-catalysts to produce active sites and reduce the activation energy for gas evolution (Maeda et al., 2005a,b, 2006). However, more work is still needed to better elucidate the effective photo-catalytic reactivity associated with the unique structure of transition metal oxides and its electronic structural interaction with (Ga_1−x_Zn_x_)(N_1−x_O_x_). The requirement of co-catalysts is not good for understanding the photo-catalytic capability and activity of (Ga_1−x_Zn_x_)(N_1−x_O_x_).

This solid solution suffers a relatively low quantum efficiency, which can be attributed to the high defect density in this solid solution. The performance of (Ga_1−x_Zn_x_)(N_1−x_O_x_) catalyst can be expected to be improved by refining the preparation method. Zinc oxide crystallizes have three forms, cubic rocksalt (RS), cubic zinc blende (ZB) and hexagonal wurtzite (WZ). The wurtzite phase has attracted much attention of researchers owing to its highest thermodynamic stability and easy synthesis (Li et al., 2020). Therefore, it becomes incrementally more challenging to obtain a mixed-phase ZnO with more than one metastable phases. In fact, mixed-phases have been predicted to be more exciting than the stable counterpart-wurtzite phase because of its unpredictable physical and chemical properties.

Hydrothermal (HT) method is commonly employed to grow ZnO single crystal with low defect density and high crystallinity. By adjusting the growth conditions, such as the pressure, temperature, time and pH, etc., ZnO crystalizes can be grown in different crystal types. In this work, two types of crystals are obtained through hydrothermal method: one of is ZnO:Ga (L) possessing multiple crystal forms and the other is ZnO:Ga (B) with wurtzite crystal structure. To the best of our knowledge, the studies on the photocatalytic performance of mixed-phase ZnO have not been reported so far. The Ga cation is octahedrally in cubic rocksalt form, and it is tetrahedrally coordinated in the zinc blende and wurtzite. The different coordinations of Ga^3+^ in the local structures can affect photocatalytic activity for their geometric and electronic configurations. Compared with wurtzite ZnO phase, ZB and RS ZnO have lower ionicity and higher crystallographic symmetry, they are expected to have the superiorities of higher carrier mobility, doping efficiency and optical gain (Ashrafi and Jagadish, 2007). Mixed phase structures in InAs polymorphs demonstrated interesting properties and futuristic applications (Caroff et al., 2009), and a synergistic effect of the homojunction formed by two coexisting phases is also observed in a photoactive nano-TiO_2_ material (Nair et al., 2011, 2012).

The photocatalytic activity of ZnO:Ga (B) and ZnO:Ga (L) was compared. Both samples were also nitrided under the same conditions, but the amount of evolved gases of ZnO:Ga (L) is much higher than that of ZnO:Ga (B) with increasing reaction time. Experimental results thus suggest that the photocatalytic activity is highly affected by doped N atoms. The effects of structural details of N-doped ZnO:Ga (L) and ZnO:Ga (B) on physicochemical characteristics and photocatalytic properties of the resulting (Ga_1−x_Zn_x_)–(N_1−x_O_x_) have been systematically investigated.

## Experimental Section

### Synthesis of ZnO:Ga(L) and ZnO:Ga (B) Bulk Crystals

ZnO bulk crystal was synthesized in the autoclave with a diameter of 90 mm and volume of 10 L under hydrothermal conditions. After compressed into tablets and sintered at 900°C for 10 h, commercial available ZnO powders and Ga_2_O_3_ powders were utilized as Ga_2_O_3_ and ZnO source (compressed 1wt% commercial available Ga_2_O_3_ powders into the ZnO tablets), and the small single ZnO crystals was suspended from the lid of the liner by a Pt wire as seeds, and KOH aqueous solution is mineralization agent. Subsequently, under our hydrothermal conditions, the bottom of autoclave is kept at 300°C and the top at 270°C for 60 days. Ga-doped ZnO single crystal was obtained via the same synthesis process. ZnGa_2_O_4_ single crystal was obtained via the same synthesis process, the only different is the small single Ga_2_O_3_ crystals were suspended as seeds.

### Preparation of Nitrided ZnO:Ga (L) and ZnO:Ga (B)

Under NH_3_ atmosphere, the ZnO:Ga (L) and ZnO:Ga (B) crystals were heated at 823 K for 6 h and then cooled naturally to 300 K.

### Characterization

The HRTEM was collected by a FEI Talos F200 S. Low temperature (5 K) PL spectra were collected by Kimmon He-Cd Laser (325 nm), where the samples were placed in a self-made liquid helium compression refrigeration system. The XRD was measured using a Bruker D8 Advance X-ray diffractometer. The EDS was conducted by an X-MaxN OXFORD Instruments. The reflectance spectra were collected using UV–VIS-NIR spectrophotometer (Shimadzu UV-3600 Plus). The photocatalytic H_2_ evolution of four ZnO:Ga samples were carried out in a homemade quartz glass reactor. In each photocatalytic test, 50 mg as-prepared photocatalysts were suspended in 25 mL aqueous solution, containing 10 mL methanol as sacrificial agents. The photocatalytic process was under continuous magnetic stirring with a 500 W mercury lamp as an excitation source (λex = 365 nm). The generated gas (0.4 mL) was intermittently extracted and measured by a gas chromatograph (GC, Shimadzu GC-2014).

## Results and Discussion

**Figure 1:**
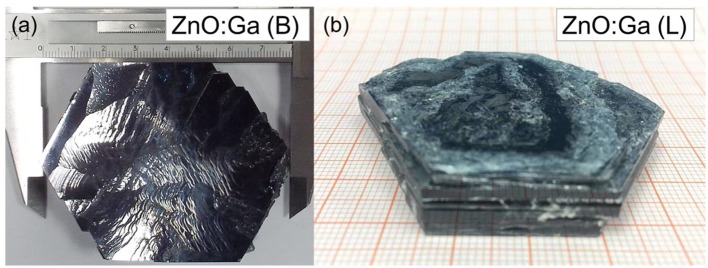
**(a)** bulk ZnO:Ga (B) and **(b)** layered ZnO:Ga (L) single crystals synthesized through hydrothermal method. The size of obtained ZnO:Ga crystals is as large as >2 inches in diameter.

**Figure 2:**
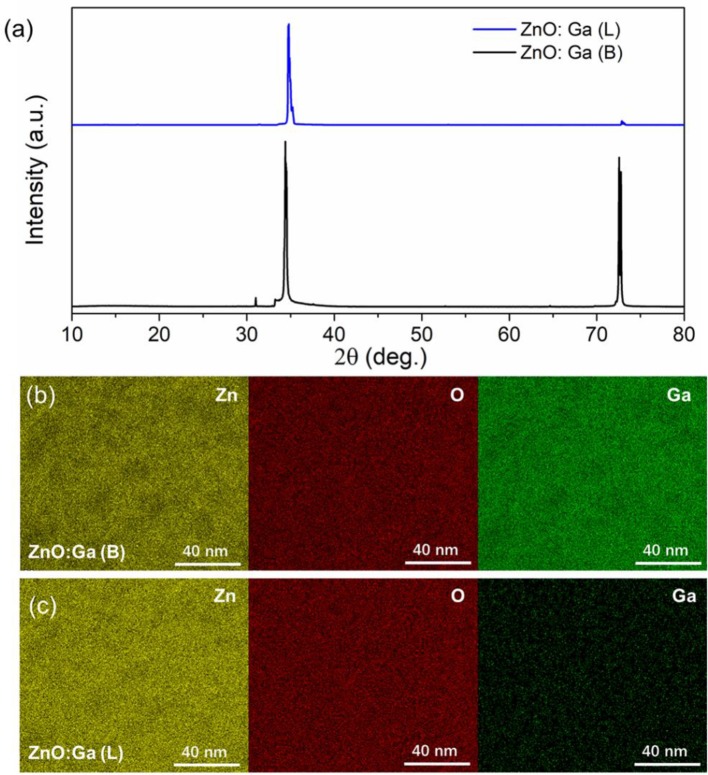
**(a)** XRD patterns of ZnO:Ga (B) and ZnO:Ga (L) single crystals; **(b)-(c)** EDS spectra for Zn, Ga, and O element mappings of ZnO:Ga (B) and ZnO:Ga (L) crystals, respectively.

**Figure 3:**
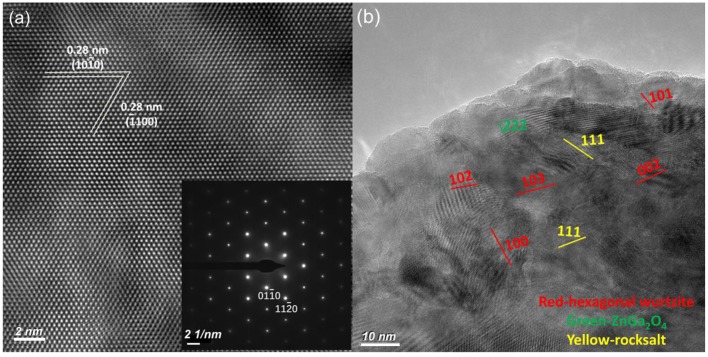
HRTEM pictures of the **(a)** bulk ZnO:Ga (B) and **(b)** layered ZnO:Ga (L) single crystals. The upper inset in **(a)** is the ideal atomic model of ZnO, and the lower one is the SAED pattern of bulk ZnO:Ga (B).

**Figure 4:**
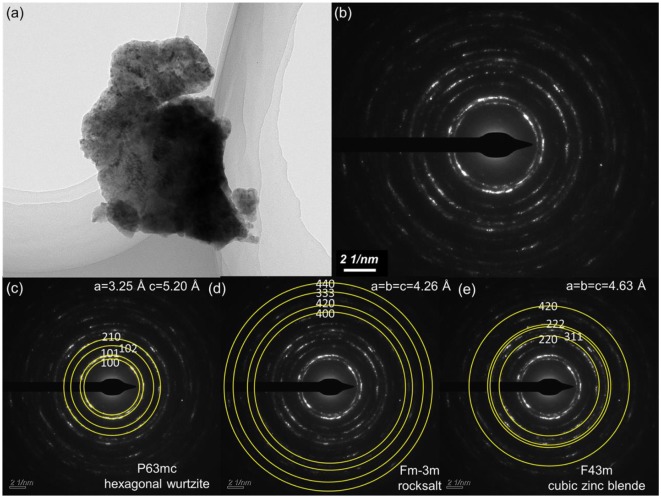
**(a)** TEM picture of a selected thin area of ZnO:Ga (L) crystal; **(b)** Corresponding SAED pattern of **(a)**; **(c–e)** marked crystal system of three different phases from ZnO:Ga (L) for **(b)**.

**Figure 5:**
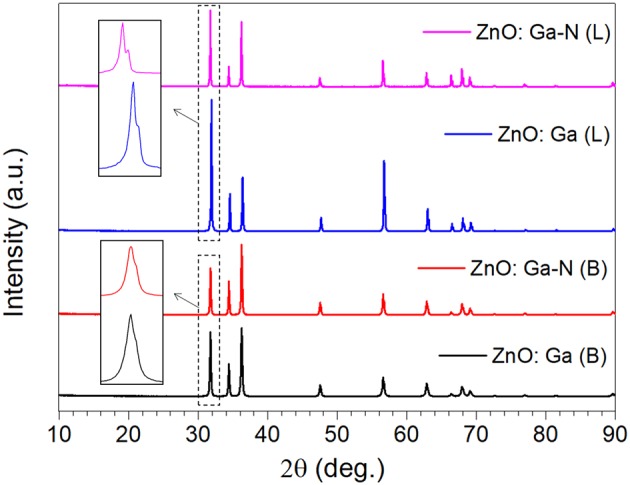
XRD patterns of the four powder samples with enlarge peaks.

Large-size ZnO crystals have been grown under hydrothermal method at the approximate thermodynamic equilibrium condition due to the comparatively low growth temperatures and the delicate transport of ZnO nutrient (Lin et al., 2009, 2011). The continuous mass transport of Zn source and stable growth rate under our synthesis condition enable to grow large-size ZnO crystals with low defect density and high crystallinity. Ga-doped ZnO crystals have been crafted through a similar method. The as-synthesized large-size ZnO:Ga (B) (dark blue, single crystal with side surface area is larger than 30 cm^2^) and ZnO:Ga (L) (dark blue, layered crystals with side surface area is larger than 20 cm^2^) and crystals are shown in Figure 1. As shown in Figure 2a, the positions of diffraction peaks of both ZnO:Ga (L) and ZnO:Ga (B) are located at the characteristic peaks of wurtzite-type ZnO. EDS analyses in Figures 2b,c also show that there is little composition difference between them. Then, HRTEM has to be used to detect the structures in them, which is undetected by powder XRD patterns. The HRTEM micrograph, shown in Figure 3a reveals that ZnO:Ga (B) has only wurtzite monophasic phase. The SAED pattern of ZnO:Ga (B) crystal (the inset in Figure 3a) shows the continuous lattice fringes of ZnO, which is in accordance with the ideal atomic model of ZnO and indicates the high single crystalline quality of ZnO:Ga (B). Figure 3b shows the lattice planes of different phases in ZnO:Ga (L). After SAED analysis, WZ, ZB and RS phases are identical and shown in Figure 4, respectively. Strategies such as doping and phase mixing enable to elevate and extend the activity of a photocatalyst in the visible range. Then, nitriding the nicely crystals under NH_3_ flow has been performed in this work to obtain (Ga_1−x_Zn_x_)(N_1−x_O_x_) crystals. It is worth noting that nitridated ZnO:Ga (L) and ZnO:Ga (B) are still crystalline (with good crystallinity), since the nitriding condition in NH_3_ atmosphere can not change its crystal form. Figure 5 shows that the diffraction peaks of nitridated ZnO: Ga (L) are shifted to lower angles (2θ) when comparing with that of ZnO: Ga (L). While the peak positions of ZnO:Ga (B) is almost unchanged when nitrided. Such difference may be attributed to the large amounts of homojunctions (in the interfaces between two different phases) and resultant oxygen vacancies inside triphasic ZnO:Ga (L), which will increase the probabilities of N doping into ZnO:Ga lattice.

**Figure 6:**
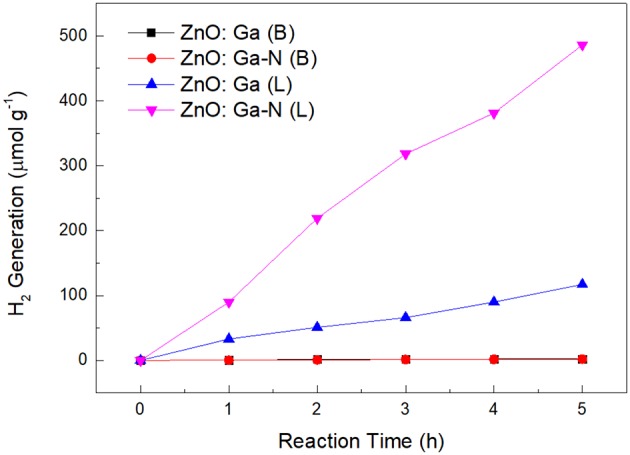
Photocatalytic properties of a typical time course of H_2_ production from water.

In terms of the kinetics of water splitting, visible light photocatalytic performance of the samples were evaluated. The rate constants were calculated by plotting H_2_ vs. time (Figure 6). As is well-known, the photocatalytic activity of photocatalysts for overall water splitting depends heavily on the particle size and crystallinity of the material (Ikeda et al., 1998; Sato et al., 2004). But investigating the factors that affecting the photocatalytic activity of soild solution (Ga_1−x_Zn_x_)(N_1−x_O_x_) is hard to be performed for the high defect densities in these materials. Our HRTEM observation revealed that ZnO:Ga (L) and ZnO:Ga (B) were nicely crystallized. Normally, the high degree of crystallization reduces amounts of defect sites significantly in the particle. In this case, sample ZnO:Ga (B) shows improved homogeneity and reduced defect densities in the materials, but they does not contribute directly to activity-enhancement. Compared to ZnO:Ga (B), sample ZnO:Ga (L) shows relatively low crystallinity and many structural imperfections. This observation is different with solid solution, and the chemical nature of solid solution might cause low mobilities of charge carriers and high recombination rates of electron-hole pairs for defect densities formed by abundant polycrystalline boundaries. But, the increased photoactivity of ZnO:Ga (L) may be attributed to the existence of homojunctions and resultant oxygen vacancies in triphasic ZnO:Ga (L), which plays an important role on efficiently separating and transporting of the photo-generated carriers, and thus avoid suffering from recombination of carriers. Considering the earlier proposed model of homojunctions in biphasic TiO_2_, the role of the three phases in separating photo-generated carriers may be explained. The intrinsic potential barrier among the phases not only restricts electrons and holes moving toward opposite directions, but also decreases the probability of recombination.

**Figure 7:**
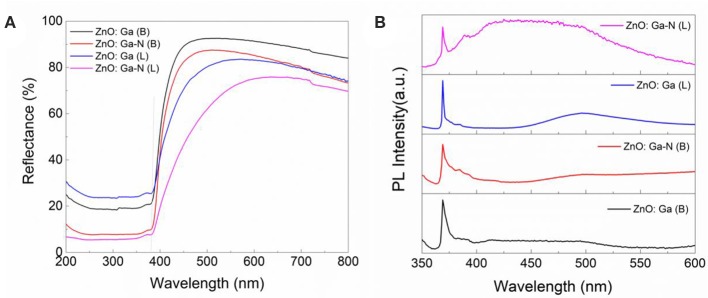
**(A)** UV-DRS, and **(B)** PL spectra (5K) of ZnO:Ga (B), N-doped ZnO:Ga (B), ZnO:Ga (L) and N-doped ZnO:Ga (L).

Comparing the ultraviolet-visible diffuse reflection spectra (UV-DRS spectrum) (Figure 7A), both the N-doped samples and ZnO:Ga (L) present red shift with respect to the ZnO:Ga (B). But the N-doped ZnO:Ga (B) sample did show little photoactivity (Figure 6). It should be pointed out that visible light absorption does not guarantee high visible light activity. Visible light activity of nitrogen doped samples is determined by the position of the defect levels in the band gap produced by nitrogen doping, and these impurity states could also lay deep inside the band gap and serve as recombination centers to significantly reduce the photo activity (Asahi and Morikawa, 2007). Photoluminescence (PL) spectra at ultralow temperature can be employed to explain the origin of the light activity of (Ga_1−x_Zn_x_)(N_1−x_O_x_) as a photocatalyst (Hirai et al., 2007). The low-temperature PL (5K) spectra of our four samples, shown in Figure 7B, have band-edge emission in the UV/blue region near 380 nm corresponding to ZnO bandgap. PL spectra of four crystals show obvious band edge emissions at 3.28 eV while no emission band at around 2.2 eV is observed (Figure 7B), demonstrating low density of deep level defects in ZnO and Ga-doped ZnO crystals. The nitrogen-doped ZnO:Ga (L) crystals not only show significant visible light photocatalytic activity but also exhibit enhanced the UV light photocatalytic activity toward the splitting of water.

As we known, the (Ga_1−x_Zn_x_) (N_1−x_O_x_) solid-solution prepared by Domen and his co-workers exhibited little photocatalytic activity (Sun et al., 2007). Because the optical band gap ZnGa_2_O_4_, GaN, ZnO is 4.4 eV, 3.4 eV and 3.2 eV, respectively, the presence of N in these structures clearly decreases the band gap below 3 eV. The origin of the ability of N-doped metal oxidates to absorb visible light is still in debate. Li et al. suggested that substitutional N doping generates a new band close to the valence band of ZnO, with which electrons from the valence band of ZnO first transfer to this new band, and then further transfer to the conductive band using visible light (Li and Haneda, 2003a,b). Initially, the origin of the visible-light absorption was thought to be the p–d repulsion between Zn 3d and N 2p electrons in the upper valence band, which results in narrowing the band gap of GaN (Maeda et al., 2005a; Maeda and Domen, 2009). Follow-up studies using PL spectroscopy and DFT calculations suggested that the visible-light absorption is caused by the electron transition from the Zn acceptor level to the conduction band, while the band-gap structure of the host GaN is maintained (Hirai et al., 2007; Yoshida et al., 2010). In the case of N-doped ZnO:Ga (L), the high activity may be explained by increase of visible light absorption and presence of empty impurity levels introduced by N doping. The empty impurity levels appear just above the valence band, and/or filled impurity levels appear just below the conduction band and these impurity levels may explain the present experimental results. This work could help to understand the origin of visible light absorption.

## Conclusions

In this work, the effects of structural details of ZnO:Ga (L) and ZnO:Ga (B) on physicochemical and photocatalytic properties of the nitriding products (Ga_1−x_Zn_x_)–(N_1−x_O_x_) have been systematically investigated. The activity of triphasic ZnO:Ga (L) systems is explained from a reduced recombination perspective mainly owing to favorable charge separation and transport restricts of the interfaces. Our experimental results clearly demonstrates that a triphasic N-doped ZnO:Ga (L) possessing WZ toghther with metastable ZB and RS phases exhibiting high visible light photocatalytic activity compared to triphasic ZnO:Ga (L) as well as N-doped wurtzite ZnO:Ga (B). The high activity of N-doped ZnO:Ga (L) may be attributed to the increase of visible light absorption and existence of empty impurity levels introduced by N doping. This work is expected to open the door for materials with several metastable states to many applications in optoelectronic, energy, environment and spintronics.

## Data Availability Statement

The datasets generated for this study are available on request to the corresponding author.

## Author Contributions

XL and FH conceived the study. XL, TL, HC, and CL performed experiments, analyzed the data, and wrote the manuscript.

## Conflict of Interest

The authors declare that the research was conducted in the absence of any commercial or financial relationships that could be construed as a potential conflict of interest.
